# Isotopically labeled sulfur compounds and synthetic selenium and tellurium analogues to study sulfur metabolism in marine bacteria

**DOI:** 10.3762/bjoc.9.108

**Published:** 2013-05-15

**Authors:** Nelson L Brock, Christian A Citron, Claudia Zell, Martine Berger, Irene Wagner-Döbler, Jörn Petersen, Thorsten Brinkhoff, Meinhard Simon, Jeroen S Dickschat

**Affiliations:** 1 Institute of Organic Chemistry, TU Braunschweig, Hagenring 30, 38106 Braunschweig, Germany; 2 Institute for Chemistry and Biology of the Marine Environment (ICBM), University of Oldenburg, Carl-von-Ossietzky-Str. 9–11, 26129 Oldenburg, Germany; 3 Helmholtz Center for Infection Research, Inhoffenstraße 7, 38124 Braunschweig, Germany; 4 Leibniz-Institut DSMZ - Deutsche Sammlung von Mikroorganismen und Zellkulturen GmbH, Inhoffenstraße 7b, 38124 Braunschweig, Germany

**Keywords:** dimethylsulfoniopropionate, *Roseobacter* clade, selenium metabolism, sulfur metabolism, volatiles

## Abstract

Members of the marine *Roseobacter* clade can degrade dimethylsulfoniopropionate (DMSP) via competing pathways releasing either methanethiol (MeSH) or dimethyl sulfide (DMS). Deuterium-labeled [^2^H_6_]DMSP and the synthetic DMSP analogue dimethyltelluriopropionate (DMTeP) were used in feeding experiments with the *Roseobacter* clade members *Phaeobacter gallaeciensis* DSM 17395 and *Ruegeria pomeroyi* DSS-3, and their volatile metabolites were analyzed by closed-loop stripping and solid-phase microextraction coupled to GC–MS. Feeding experiments with [^2^H_6_]DMSP resulted in the incorporation of a deuterium label into MeSH and DMS. Knockout of relevant genes from the known DMSP demethylation pathway to MeSH showed in both species a residual production of [^2^H_3_]MeSH, suggesting that a second demethylation pathway is active. The role of DMSP degradation pathways for MeSH and DMS formation was further investigated by using the synthetic analogue DMTeP as a probe in feeding experiments with the wild-type strain and knockout mutants. Feeding of DMTeP to the *R. pomeroyi* knockout mutant resulted in a diminished, but not abolished production of demethylation pathway products. These results further corroborated the proposed second demethylation activity in *R. pomeroyi*. Isotopically labeled [^2^H_3_]methionine and ^34^SO_4_^2−^, synthesized from elemental ^34^S_8_, were tested to identify alternative sulfur sources besides DMSP for the MeSH production in *P. gallaeciensis*. Methionine proved to be a viable sulfur source for the MeSH volatiles, whereas incorporation of labeling from sulfate was not observed. Moreover, the utilization of selenite and selenate salts by marine alphaproteobacteria for the production of methylated selenium volatiles was explored and resulted in the production of numerous methaneselenol-derived volatiles via reduction and methylation. The pathway of selenate/selenite reduction, however, proved to be strictly separated from sulfate reduction.

## Introduction

**Figure 1:**
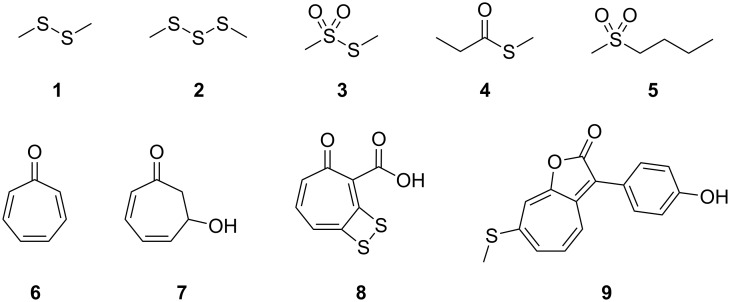
*Roseobacter* clade metabolites.

The *Roseobacter* clade within the class of alphaproteobacteria is found both in seawater and marine sediments and occurs often in association with marine algae [1]. Genome data from sequenced clade members revealed that pathways for the degradation of aromatic compounds and sulfur metabolic pathways are widespread [2]. This is reflected by their volatile bouquets that are dominated by sulfur compounds such as polysulfides **1** and **2** (Figure 1), thiosulfonates **3**, thioesters **4**, or sulfones **5**, and phenylacetate-derived volatiles such as the moderately antibacterial compounds tropone (**6**) and tropone hydrate **7** [3–6]. The cooperation of phenylacetate degradation and sulfur metabolism is manifested in the production of the antibiotic tropodithietic acid (TDA, **8**) [7–8] and the roseobacticides, a class of algicides, represented by, e.g., roseobacticide A (**9**) [9–10].

The high abundance of sulfur volatiles throughout the *Roseobacter* clade raises the question about the nature of their sulfur sources. The most important organic sulfur metabolite in marine environments is dimethylsulfoniopropionate (DMSP), which is produced by a wide range of marine organisms and in especially large amounts by dinoflagellates [11]. DMSP is degraded by marine bacteria either under the formation of methanethiol (MeSH) or of dimethyl sulfide (DMS) with a large impact on both the global sulfur cycle and climate [12–13].

**Scheme 1:**
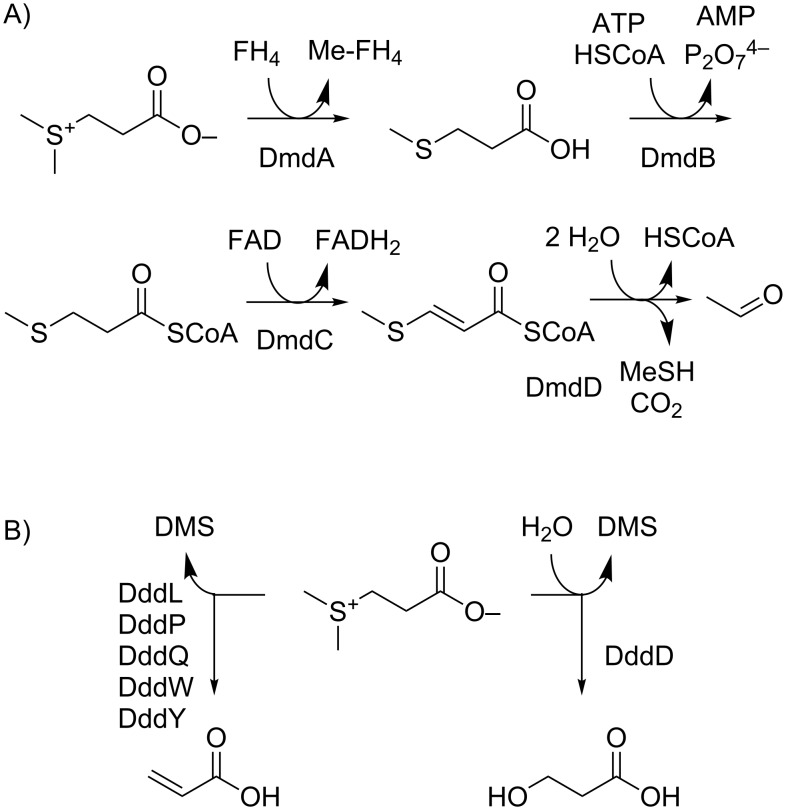
Degradation of DMSP via (A) demethylation pathway and (B) cleavage pathways. FH_4_: tetrahydrofolate.

DMSP degradation to MeSH starts with the DmdA mediated demethylation to 3-(methylthio)propionate (Scheme 1A) [14–15] followed by its conversion into its CoA-thioester by DmdB and oxidation by the FAD-dependent dehydrogenase DmdC. The addition of water to 3-(methylthio)acryloyl-CoA by the enoyl-CoA hydratase DmdD results in a hemithioacetal, which collapses under release of acetaldehyde, carbon dioxide and MeSH [16]. Several enzymes for the cleavage of DMS from DMSP (Scheme 1B) have been described [11]. DddD catalyzes the hydrolysis of DMSP to 3-hydroxypropionate and DMS, while five different classes of enzymes (DddP, DddL, DddQ, DddW, DddY) have been identified for the lysis of DMSP to acrylate and DMS.

**Scheme 2:**
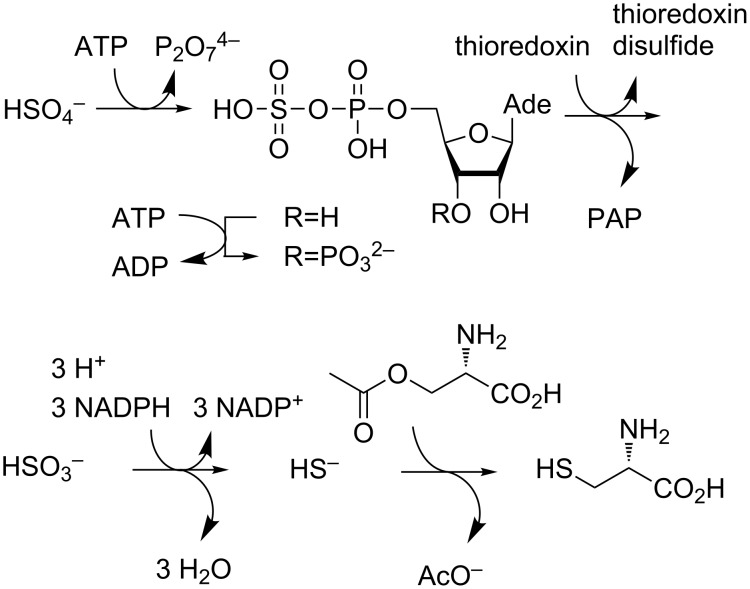
Sulfate reduction pathway and incorporation of sulfur into the amino acid pool. PAP: adenosine 3’,5’-bisphosphate.

A second obvious candidate as a source for sulfur volatiles is inorganic sulfate, which can be reduced by *Roseobacter* clade members to hydrogen sulfide via adenylyl sulfate, 3’-phosphoadenylyl sulfate, and sulfite (Scheme 2) [17]. Hydrogen sulfide enters the amino acid pool by reaction with *O*-acetyl-L-serine to L-cysteine. Elemental sulfur, hydrogen sulfide, or thiosulfate can be funneled via the lithotrophic sulfur oxidation (Sox) pathway to sulfate [18–22].

Analogous degradation steps for the selenium and tellurium derivatives of DMSP (dimethylseleniopropionate, DMSeP, and dimethyltelluriopropionate, DMTeP) and the reduction of selenate, selenite, tellurate, and tellurite may be possible via the described pathways. However, in *Desulfovibrio desulfuricans* subsp. *aestuarii* only minor amounts of selenate are reduced via the sulfate reduction pathway [23]. Instead, a special selenate reductase SerABC for selenate reduction to selenite was characterized from *Thauera selenatis* [24].

The effective degradation of DMSP by members of the *Roseobacter* clade via the demethylation pathway to MeSH was demonstrated in a previous study using a closed-loop stripping apparatus (CLSA) for the trapping of volatiles on charcoal [25]. The substrate scope of the involved enzymatic machinery was tested in feeding experiments with DMSeP, which is produced by the salt marsh cordgrass *Spartina alternifolia* amended with selenate [26], and the non-natural analogue DMTeP. DMSeP was effectively metabolized to give methaneselenol-derived volatiles, whereas the verification of tellurium-containing volatiles from DMTeP remained elusive [25].

Here we report the results of an in-depth investigation of sulfur metabolic pathways to volatile sulfur compounds in marine alphaproteobacteria by feeding of isotopically labeled sulfur compounds and synthetic selenium and tellurium analogues to wildtype and relevant mutant strains. A reinvestigation of DMTeP conversion by marine bacteria from the *Roseobacter* clade into methylated tellurium volatiles using a modified analytical technique is also presented.

## Results and Discussion

### Usage of [^2^H_6_]DMSP and DMTeP as synthetic probes to study DMSP degradation pathways in marine bacteria

**Figure 2:**
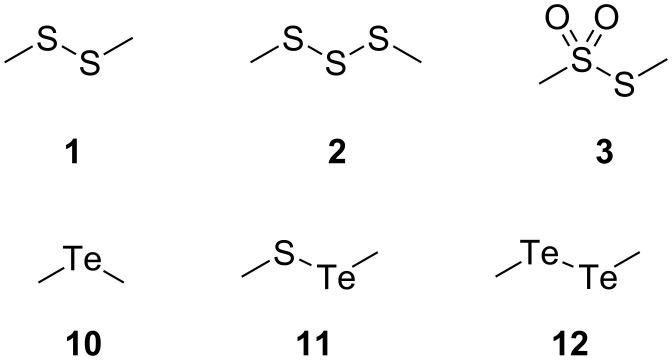
Volatiles from *P. gallaeciensis* DSM 17395 and *R. pomeroyi* DSS-3. Feeding of [^2^H_6_]DMSP results in deuterium incorporation into compounds **1**–**3**, feeding of DMTeP results in the formation of compounds **10**–**12**.

**Figure 3:**
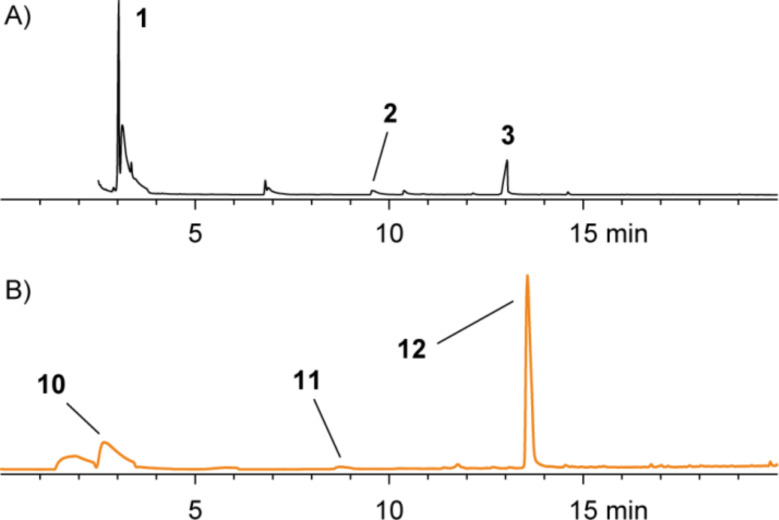
Chromatograms of headspace extracts from *P. gallaeciensis* DSM 17395 after feeding of DMTeP by the use of (A) CLSA (black) and (B) SPME (orange).

**Figure 4:**
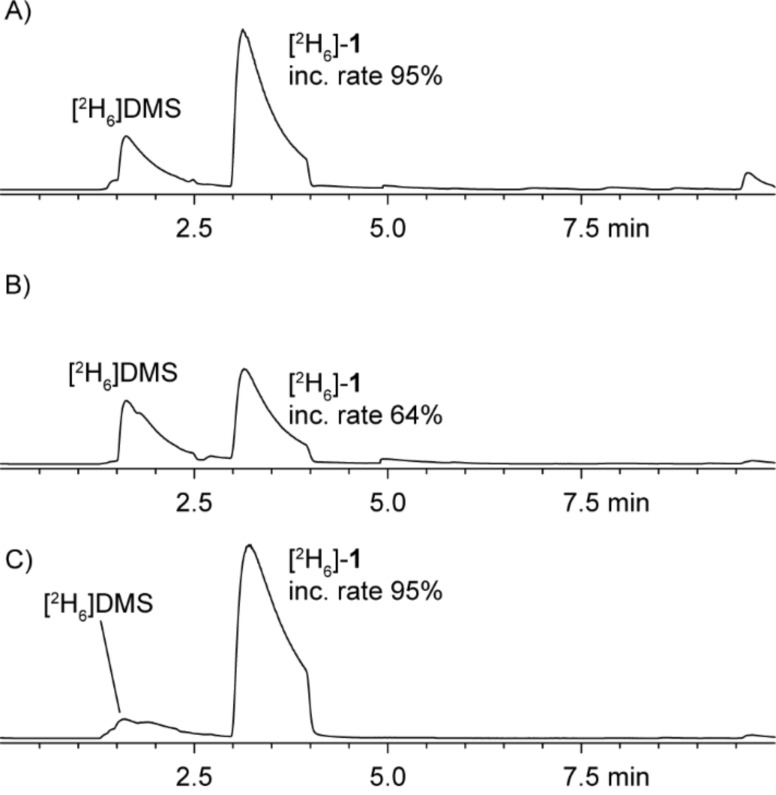
Chromatograms of headspace extracts obtained after feeding of [^2^H_6_]DMSP by the use of SPME from (A) *R. pomeroyi* DSS-3 wild type, (B) *R. pomeroyi* DSS-3 *dmdA*^−^, and (C) *R. pomeroyi* DSS-3 *dddQ*^−^. Trace amounts of [^2^H_6_]-**2** and [^2^H_6_]-**3** were also found (not shown).

**Figure 5:**
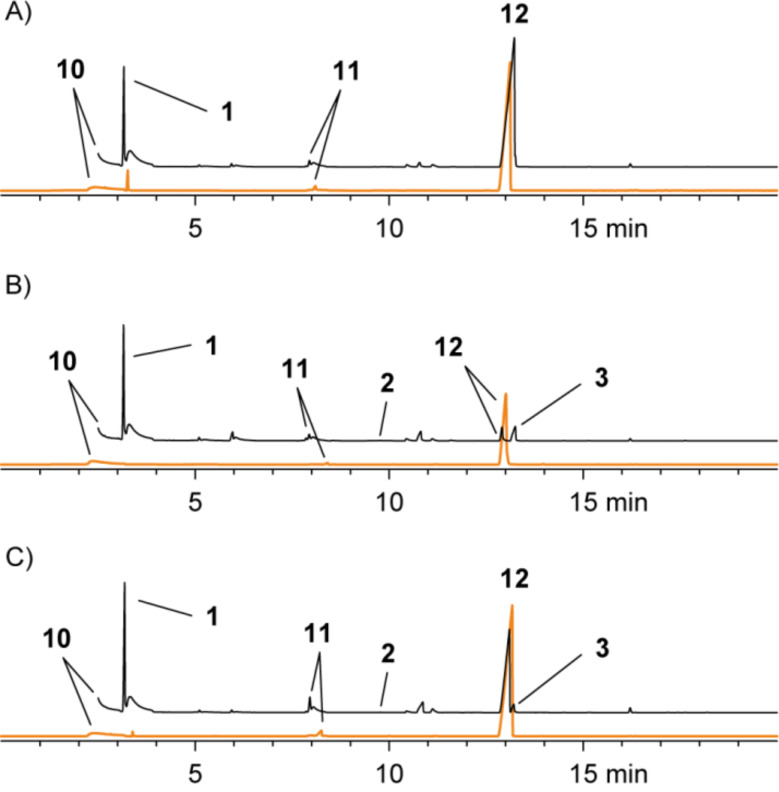
Chromatograms of headspace extracts from (A) *R. pomeroyi* DSS-3 wild type, (B) *R. pomeroyi* DSS-3 *dmdA*^−^, and (C) *R. pomeroyi* DSS-3 *dddQ*^−^ after feeding of DMTeP by use of CLSA (black) and SPME (orange).

The volatiles released by agar-plate cultures of *Phaeobacter gallaeciensis* DSM 17395 and *Ruegeria pomeroyi* DSS-3 strains grown on half-strength MB2216 medium supplemented with [^2^H_6_]DMSP or DMTeP were collected by solid-phase microextraction (SPME) and by CLSA. The obtained headspace extracts were subsequently analyzed by GC–MS, leading to the identification of the volatiles dimethyl disulfide (**1**), dimethyl trisulfide (**2**), *S*-methyl methanethiosulfonate (**3**), dimethyl telluride (**4**), dimethyl telluryl sulfide (**5**), and dimethyl ditelluride (**6**), which are shown in Figure 2. Representative chromatograms are depicted in Figures 3–5.

### Investigating DMSP degradation pathways in *Phaeobacter gallaeciensis* DSM 17395

Based on the genetic information, *P. gallaeciensis* should be able to perform both DMSP degradation pathways, as a *dmd*-gene cluster for the demethylation pathway is located on a 262 kb plasmid and a gene for the lyase DddP is encoded on its chromosome. Isotopically labeled [^2^H_6_]DMSP was efficiently incorporated into methanethiol-derived sulfur volatiles like dimethyl disulfide (**1**), dimethyl trisulfide (**2**), and *S*-methyl methanethiosulfonate (**3**) by *P. gallaeciensis* (incorporation rate 91%). Knockout of the *dmdA*-gene significantly diminished this incorporation rate (27%). The ratio of DMS production was increased from below 0.05% of the total sulfur-containing volatile material in the wild type strain to about 2% in the *dmdA* knockout mutant. The decreased incorporation rate and the relatively higher amounts of the DMSP lysis product DMS can be explained with the blockage of the *dmd* demethylation pathway. To rule out a spontaneous decomposition of DMSP to 3-(methylthio)propionate that would serve as a substrate for *dmd*-gene-mediated liberation of MeSH even in the absence of an active *dmdA* gene, [^2^H_6_]DMSP was fed to a knockout mutant with a loss of the 262 kb plasmid [26]. This plasmid-cured strain exhibited the same reduced incorporation rates (32%) of deuterium label into the MeSH-derived volatiles. The residual demethylation activity may be due to other as yet unidentified enzymes involved in the liberation of MeSH from DMSP that can, however, not be located on the 262 kb plasmid. Consistent with previous results [25], after feeding of DMTeP to *P. gallaeciensis* no tellurium compounds were detected in the CLSA headspace extracts (Figure 3A), although a strong metallic to garlic-like smell as typical for organotellurium compounds evolved from the agar-plate cultures. However, SPME extracts of *P. gallaeciensis* cultures amended with the same amount of DMTeP and grown under identical conditions as in the CLSA-based experiment were dominated by dimethyl ditelluride (**12**), accompanied by dimethyl telluryl sulfide (**11**) and dimethyl telluride (**10**, Figure 3B). This demonstrated that the SPME method is more sensitive for the analysis of organotellurium compounds than is CLSA. Compound **12** can arise by oxidative dimerization of the unnatural demethylation pathway product methanetellurol, whereas **11** is the cross-coupling product of methanetellurol and methanethiol that is also formed during growth on MB2216 medium alone [25]. In contrast to DMSP and DMSeP, which are degraded by *P. gallaeciensis* mainly via the demethylation pathway (>99%) [25], a significant contribution of the cleavage pathway (21% by peak integration) was observed for the degradation of DMTeP.

### Investigating DMSP degradation pathways in *Ruegeria pomeroyi* DSS-3 (DSM 15171^T^)

Genes for three DMSP lyases (*dddP*, *dddQ*, and *dddW*) and for the demethylation pathway (*dmdA*–*D*) are encoded in the genome of *R. pomeroyi* DSS-3. Feeding of [^2^H_6_]DMSP to this strain led to its degradation via the demethylation pathway (90%) and the cleavage pathway (10%). This ratio of the two pathways was determined by comparison of the peak areas in the gas chromatogram of SPME extracts (Figure 4A, the peak area of the MeSH dimer **1** has to be considered twice; compounds **2** and **3** also originating from MeSH were not considered, because they were only produced in trace amounts). A reduced demethylation activity was observed by feeding of [^2^H_6_]DMSP to a *dmdA*^−^ knockout mutant strain of DSS-3. The incorporation rates of the deuterium labeling into the MeSH-derived volatiles were reduced (64% for **1**) in comparison to the wild type strain (95%) and the ratio of the cleavage pathway increased to 35% (Figure 4B). The incorporation of isotopic labeling from [^2^H_6_]DMSP by the *dmdA*^−^ mutant strongly suggests that also in *R. pomeroyi* a second pathway for DMSP demethylation is present, which is in contrast to previous reports [14]. Knockout of *dddQ* gave the opposite result and decreased the ratio of the cleavage pathway to 1%, demonstrating its premier responsibility for DMSP lysis in the concert of the three lyases in *R. pomeroyi* (Figure 4C).

Feeding of DMTeP to the *R. pomeroyi* wild type strain resulted in the production of large amounts of the demethylation pathway product **12** besides minor amounts of **11** and the lysis product dimethyl telluride (**10**; Figure 5). The total amounts of organotellurium volatiles were much higher than those observed for *P. gallaeciensis*, and therefore, even the CLSA technique that proved to be not suitable to collect tellurium volatiles from *P. gallaeciensis* cultures grown on DMTeP was in this case successfully applied. The *R. pomeroyi dmdA*^−^ knockout mutant showed significantly reduced production of **11** and **12**, but small residual amounts likely evolving via the second unidentified demethylation pathway could still be detected. Knockout of the gene encoding for the DMSP lyase *dddQ* had no effect on the production of **10**. This is in sharp contrast to the observations made with [^2^H_6_]DMSP, implicating a rather narrow substrate specificity for DMSP of DddQ. One or both of the remaining two lyases DddP and DddW may be responsible for the strain’s capability to lyse DMTeP. The potential of DddP for DMTeP lysis has already been evaluated in *P. gallaeciensis* whose genome only encodes DddP as a single DMSP lyase, while no conclusions can be drawn on the participation of DddW.

### Exploring the source of methanethiol

**Scheme 3:**
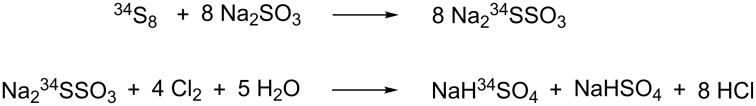
Synthesis of ^34^S-labeled thiosulfate and sulfate.

Two possible sources for MeSH production from MB2216 medium are sulfate via reduction to sulfide and methylation, or methionine via γ-lysis. The participation of both mechanisms was explored by feeding of isotopically labeled sulfate, thiosulfate and methionine. The volatiles released by agar-plate cultures of *P. gallaeciensis* fed with NaH^34^SO_4_ or Na^34^SSO_3_, both synthesized from elemental ^34^S_8_ (Scheme 3), were collected by using a CLSA and analyzed by GC–MS. No incorporation of the ^34^S-labeling from sulfate or thiosulfate into the MeSH-derived volatiles was observed, pointing strongly away from a significant involvement of sulfate reduction in the biosynthesis of MeSH. Instead, feeding of [*methyl*-^2^H_3_]methionine led to an effective incorporation of the deuterium labeling into **1** (85% incorporation rate, Figure S1 of Supporting Information File 1) and other MeSH-derived volatiles.

### Selenium volatiles from marine alphaproteobacteria

**Figure 6:**
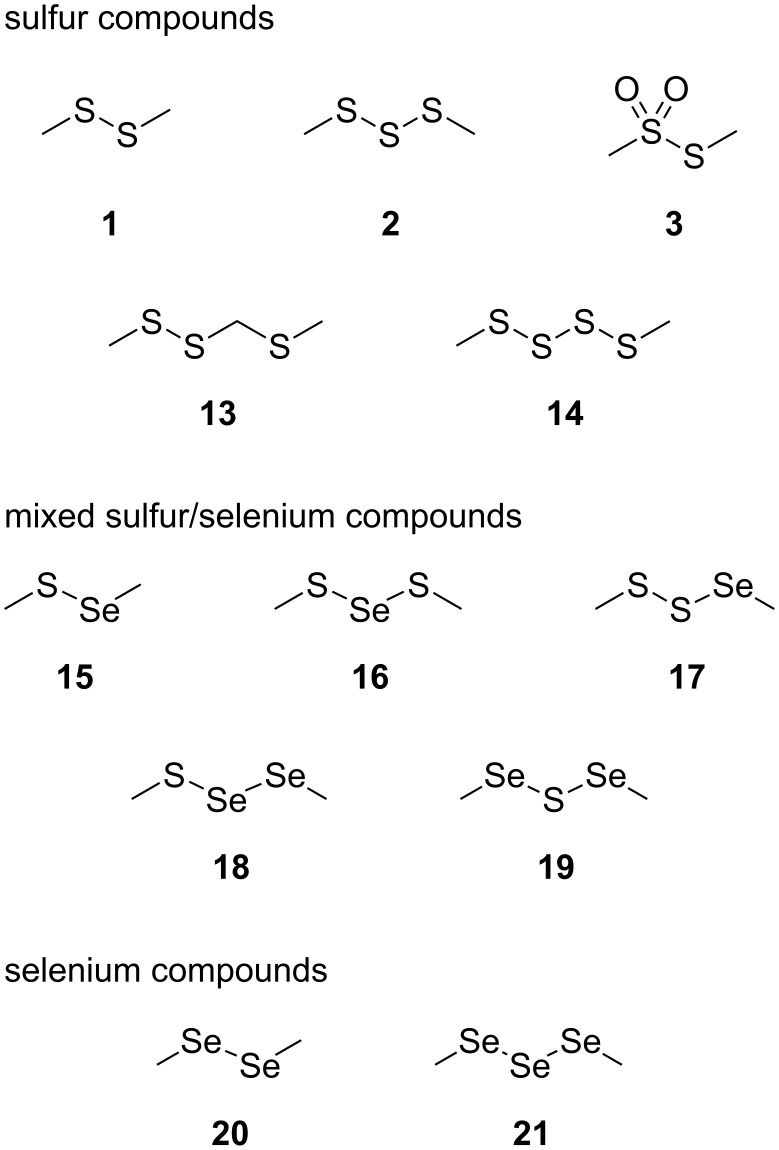
Volatiles from *P. gallaeciensis* after feeding of selenate and selenite.

**Figure 7:**
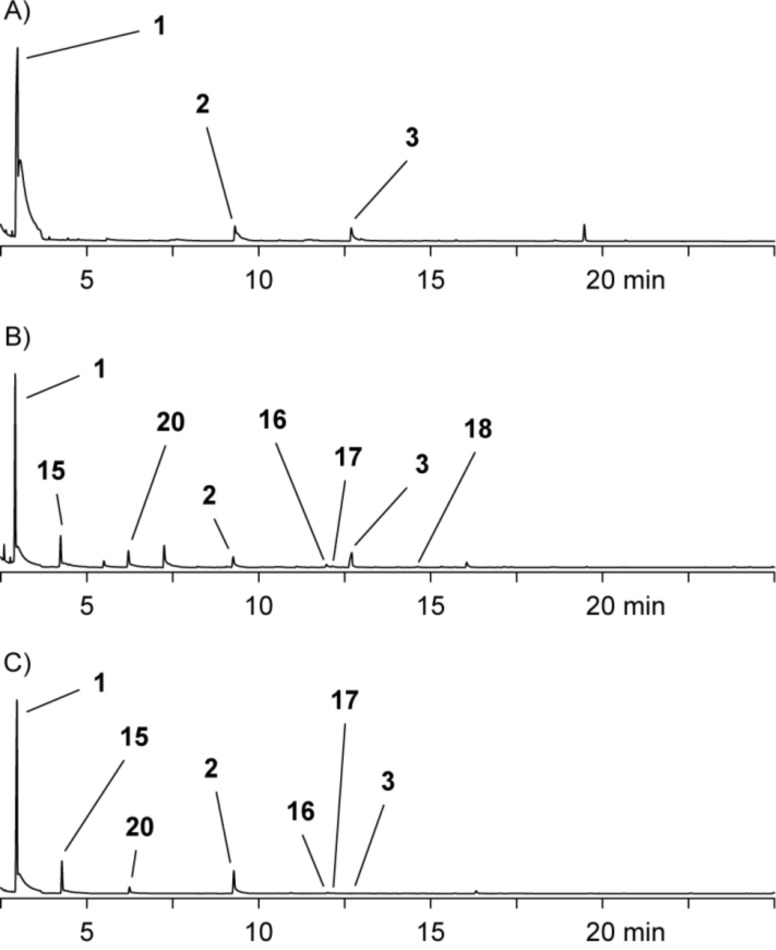
Chromatograms of headspace extracts from *P. gallaeciensis* grown on (A) 50% MB2216, (B) 50% MB2216 + 1 mmol/l Na_2_SeO_4_, (C) 50% MB2216 + 1 mmol/l Na_2_SeO_3_.

Agar-plate cultures of *P. gallaeciensis* grown on half-strength MB2216 medium, plain or amended with different selenium salts, were analyzed with the CLSA technique. A variety of sulfur and selenium-containing volatiles was identified, as summarized in Figure 6. The respective gas chromatograms are shown in Figure 7.

Feeding of Na_2_SeO_4_ to *P. gallaeciensis* resulted in the formation of a number of selenium volatiles including dimethyl diselenide (**20**) and dimethyl triselenide (**21**). In addition, the mixed sulfur/selenium compounds dimethylselenyl sulfide (**15**), bis(methylthio) selenide (**16**), methylseleno disulfide (**17**), methyl methylthio diselenide (**18**), and bis(methylseleno) sulfide (**19**) were found. A similar headspace composition was observed in the feeding experiment with Na_2_SeO_3_. All selenium compounds are derived from methaneselenol, and their formation by *P. gallaeciensis* has previously been observed in feeding experiments with dimethylseleniopropionate [25]. The participation of the sulfate reduction pathway in the reduction of selenate and selenite to methaneselenol was tested by knockout of the genes for the bifunctional sulfate adenylyltransferase/adenylylsulfate kinase (*cysC*, strain WP73, locus tag PGA1_c24800) and the sulfite reductase (*cysI*, strain WP45, locus tag PGA1_c20760). No significant differences of the volatile profiles were observed in feeding experiments with the mutant strains compared to the wild type, indicating that as yet unidentified enzymes are responsible for the selenate/selenite reduction in *P. gallaeciensis*. These respective genes are not located on the 262 kb plasmid pPGA1_262, as the plasmid-cured mutant strain [27] was still able to produce methylated selenium volatiles from selenate and selenite.

**Figure 8:**
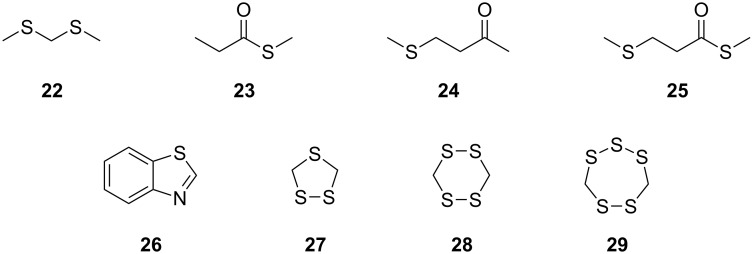
Additional sulfur volatiles.

The same sulfur and selenium volatiles were also found in the CLSA headspace analyses with agar plate cultures of *Roseobacter denitrificans* DSM 7001^T^, *Oceanibulbus indolifex* DSM 14862^T^, *Dinoroseobacter shibae* DSM 16493^T^, *Labrenzia alexandrii* DFL-11^T^, *Roseovarius mucosus* DFL-24^T^, and *Hoeflea phototrophica* DFL-43^T^ (Supporting Information File 1, Figure S2). The production of methylated selenium volatiles from the selenate and selenite salts was especially pronounced for *H. phototrophica* from the order rhizobiales, a close relative of *Roseobacter* clade bacteria. In addition, the sulfur volatiles bis(methylthio)methane (**22**), *S*-methyl propanethioate (**23**), 4-(methylthio)butan-2-one (**24**), *S*-methyl 3-(methylthio)propanethioate (**25**), and benzothiazole (**26**) were found in strain-specific patterns (Figure 8). Cyclic sulfur volatiles, such as 1,2,4-trithiolane (**27**), 1,2,4,5-tetrathiane (**28**), and lenthionine (**29**), compounds that are known from Shiitake mushrooms (*Lentinus edodes*) [28–30], were solely produced by *R. denitrificans*.

## Conclusion

In summary, we have shown that isotopically labeled natural sulfur compounds and their (synthetic) selenium and tellurium analogues can be applied in feeding experiments that give valuable insights into the sulfur metabolic pathways of marine bacteria. Deuterated [^2^H_6_]DMSP and its artificial analogue DMTeP can be transformed by lysis into [^2^H_6_]dimethyl sulfide and dimethyl telluride, or via the demethylation pathway to [^2^H_3_]MeSH and methanetellurol, respectively. Knockout of *dmdA* in *P. gallaeciensis* and *R. pomeroyi* demonstrated its major involvement in the demethylation pathway, but residual demethylation activity was observed suggesting the presence of a second DMSP demethylation pathway. Further experiments, preferably in vitro with the different classes of purified DMSP lyases, will be required to investigate their participation in DMTeP lysis. On full medium, containing both methionine and sulfate, feeding experiments with [*methyl*-^2^H_3_]methionine demonstrated that methionine γ-lysis is an important pathway for MeSH production in *P. gallaeciensis*. Sulfate reduction and methylation was not observed in feeding experiments with ^34^S-labeled sulfate or thiosulfate, likely because sulfate reduction was not active under the experimental growth conditions. This is in sharp contrast to the observations that large amounts of methaneselenol-derived volatiles are released during growth of *P. gallaeciensis* and several other marine bacteria on sodium selenate, also requiring reduction and methylation. These observations suggest that the formation of volatiles from selenate is a detoxification mechanism that is strictly separated from sulfate reduction, as was confirmed by investigation of a *P. gallaeciensis* knockout mutant impaired in sulfate reduction. This strict pathway separation may help to avoid incorporation of toxic selenium into other sulfur metabolic pathways such as sulfur amino acid metabolism. Future experiments in our laboratory will be performed to identify the genes involved in selenate reduction in marine bacteria.

## Experimental

**Strains and culture conditions:** A complete list of all wild type and mutant strains used in this study is given in Supporting Information File 1, Table S1. *Phaeobacter gallaeciensis* DSM 17395 [17], *Ruegeria pomeroyi* DSS-3 (DSM 15171^T^) [31], *Roseobacter denitrificans* OCh 114 (DSM 7001^T^) [32], *Oceanibulbus indolifex* HEL-45 (DSM 14862^T^) [33], *Dinoroseobacter shibae* DFL-12 (DSM 16493^T^) [34], *Labrenzia alexandrii* DFL-11 (DSM 17067^T^) [35], *Hoeflea phototrophica* DFL-43 (DSM 17068^T^) [36], and *Roseovarius mucosus* DFL-24 (DSM 17069^T^) [37] were precultured in half-strength marine broth (peptone (2.5 g/L), yeast extract (0.5 g/L), Fe(III) citrate (0.05 g/L), NaCl (9.725 g/L), MgCl_2_ (2.95 g/L), Na_2_SO_4_ (1.62 g/L), CaCl_2_ (0.9 g/L), KCl (0.275 g/L), NaHCO_3_ (0.08 g/L), KBr (0.04 g/L), SrCl_2_ (17 mg/L), H_3_BO_3_ (11 mg/L), Na-silicate (2 mg/L), NaF (1.2 mg/L), (NH_4_)NO_3_ (0.8 mg/L), Na_2_HPO_4_ (4 mg/L); pH 7.6) at 28 °C with shaking (160 rpm). When necessary, kanamycin sulfate (60 μg/mL) was added after autoclaving.

**Feeding experiments, sampling and GC–MS analyses:** Feeding experiments with [^2^H_6_]DMSP and DMTeP, sampling by CLSA or SPME (Supelco Analytical, Bellefonte, USA; 75 μm carboxen/polydimethylsiloxane) and GC–MS analyses were carried out as described previously [24]. For the feeding experiments with ^34^S-labeled thiosulfate or sulfate, sodium sulfate deficient half-strength marine broth agar medium was amended after autoclavation with 2.9 mmol/l Na_2_^34^SSO_3_ (50% ^34^S-enrichment) or 5.7 mmol/l NaH^34^SO_4_ (25% ^34^S-enrichment). For the feeding experiments with [*methyl*-^2^H_3_]methionine, selenate, and selenite, half-strength marine broth agar medium was supplemented after autoclaving with 1 mmol/l of [*methyl*-^2^H_3_]methionine, Na_2_SeO_3_, and Na_2_SeO_4_, respectively.

**Construction of *P. gallaeciensis* strain CZ01:** Primers used in this study are listed in Supporting Information File 1, Table S2. For the gene disruption of *dmdA* a 3.6 kb chromosomal fragment containing the gene was amplified with the primers Cl1f and Cl1r. The amplified product was ligated into the *Eco*RV site of pBluescript KS(+). The kanamycin resistance gene (amplified from pBBR1MCS-2 using the primers nptII-f and nptII-r) was cloned into the *Eco*RV site of the previously obtained plasmid that already carried the *dmdA* gene region. *P. gallaeciensis* DSM 17395 was transformed by electroporation with this plasmid, leading to the strain CZ01 (*dmdA*::*kan*), which was ratified by PCR.

**General synthetic methods:** Chemicals were purchased from Acros Organics (Geel, Belgium) or Sigma-Aldrich Chemie GmbH (Steinheim, Germany), and used without further purification. ^34^S_8_ (99.93% enriched) was purchased from Campro Scientific GmbH (Berlin, Germany). Infrared spectra were recorded on a Bruker Tensor 27 ATR spectrometer.

**Na_2_^34^SSO_3_·5H_2_O:** Elemental ^34^S_8_ (0.800 g, 23.5 mmol, 1.0 equiv) was added to a solution of Na_2_SO_3_ (2.97 g, 23.6 mmol, 1.0 equiv) in water (40 mL) and stirred under reflux until the sulfur was consumed (48 h). Filtration and concentration in vacuo gave Na_2_^34^SSO_3_·5H_2_O (5.90 g, 23.6 mmol, quant.) as a colorless solid. IR (ATR): ν = 1133 (s), 1008 (s), 674 (s), 539 (w) cm^−1^.

**NaH^34^SO_4_:** To an aqueous HCl solution (6 m, 400 mL) was added a saturated KMnO_4_ solution (400 mL), and the emerging Cl_2_ was condensed in a second flask at −40 °C. By slow warming to room temperature the evolving Cl_2_ was funneled through a solution of Na_2_^34^SSO_3_·5H_2_O (3.11 g, 12.4 mmol, 1.0 equiv) in water (50 mL). Filtration and concentration in vacuo gave NaH^34^SO_4_ (1.25 g, 10.3 mmol, 83%, 50% ^34^S-enchrichment) as a colorless solid. IR (ATR): ν = 1160 (s), 1011 (m), 904 (m), 607 (m), 576 (m), 494 (w) cm^−1^.

## Supporting Information

File 1 Tables with strains, primers and the full results of the headspace analyses.
