# Supplementary material for: Isotopically labeled sulfur compounds and synthetic selenium and tellurium analogues to study sulfur metabolism in marine bacteria
Source: Beilstein J Org Chem. 2013 May 15;9:942–50. doi: 10.3762/bjoc.9.108 (PMC3678758; doi:10.3762/bjoc.9.108)
Supplement: File 1 — Tables with strains, primers and the full results of the headspace analyses. [file Beilstein_J_Org_Chem-09-942-s001.pdf]

Supporting Information

for

Isotopically labeled sulfur compounds and synthetic selenium and tellurium analogues to study sulfur metabolism in marine bacteria

Nelson L. Brock[1], Christian A. Citron[1], Claudia Zell[1,2], Martine Berger[2], Irene Wagner-Döbler[3], Jörn Petersen[4], Thorsten Brinkhoff[2], Meinhard Simon[2], and Jeroen S. Dickschat[1*]

Address: [1]Institute of Organic Chemistry, TU Braunschweig, Hagenring 30, 38106 Braunschweig, Germany, [2]Institute for Chemistry and Biology of the Marine Environment (ICBM), University of Oldenburg, Carl-von-Ossietzky-Str. 9–11, 26129 Oldenburg, Germany, and [3]Helmholtz Center for Infection Research, Inhoffenstraße 7, 38124 Braunschweig, Germany and [4]Leibniz-Institut DSMZ - Deutsche Sammlung von Mikroorganismen und Zellkulturen GmbH, Inhoffenstraße 7b, 38124 Braunschweig, Germany.

Email: Jeroen Sidney Dickschat – j.dickschat@tu-bs.de

* Corresponding author

Tables with strains, primers and the full results of the headspace analyses

Table S1: Wild type and mutant strains investigated in this study.

Strain	Genotype	Locus Tag[a]	Source
Phaeobacter gallaeciensis			
DSM 17395	wild type		[1]
CZ01	*dmdA*::*kan*	PGA1_262p01830	This work
WP45	*cysI*::EZTn5	PGA1_c20760	[1]
WP73	*sat/cysC*::EZTn5	PGA1_c24800	[1]
262 kb mutant	DSM 17395 Δ262 kb		[2]
Ruegeria pomeroyi			
DSS-3 (= DSM 15171^T)	wild type		[3]
J471	*dmdA*::pBIO1870	SPO1913	[4]
J474	*dddQ*::pBIO1869	SPO1596	[5]
Roseobacter denitrificans			
OCh 114 (= DSM 7001^T)	wild type		[6]
Oceanibulbus indolifex			
HEL-45 (= DSM 14862^T)	wild type		[7]
Dinoroseobacter shibae			
DFL-12 (= DSM 16493^T)	wild type		[8]
Labrenzia alexandrii			
DFL-11 (= DSM 17067^T)	wild type		[9]
Hoeflea phototrophica			
DFL-43 (= DSM 17068^T)	wild type		[10]
Roseovarius mucosus			
DFL-24 (= DSM 17069^T)	wild type		[11]

[a] Locus tags of the targeted genes.

Table S2: Oligonucleotide primers used in this study.

Primer	Sequence
Cl1f	ATTGGAACTGGCAAGTGCGGC
Cl1r	AACTTCCATCGTGACTATGTCAGC
nptII-f	CCTTTAGTGAGGGTTAATTGCGC
nptII-r	GGATGAATGTCAGCTACTGG

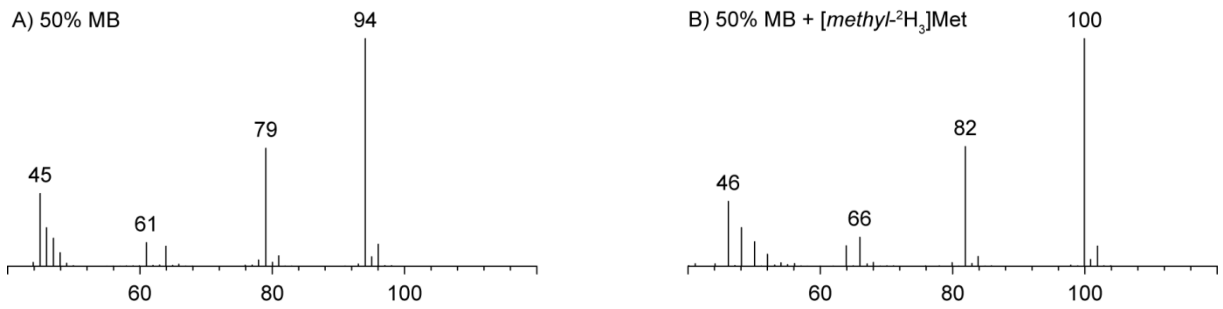

Figure S1: Incorporation of [*methyl*-$^2H_3$]methionine into dimethyl disulfide (**1**) by *Phaeobacter gallaeciensis*.

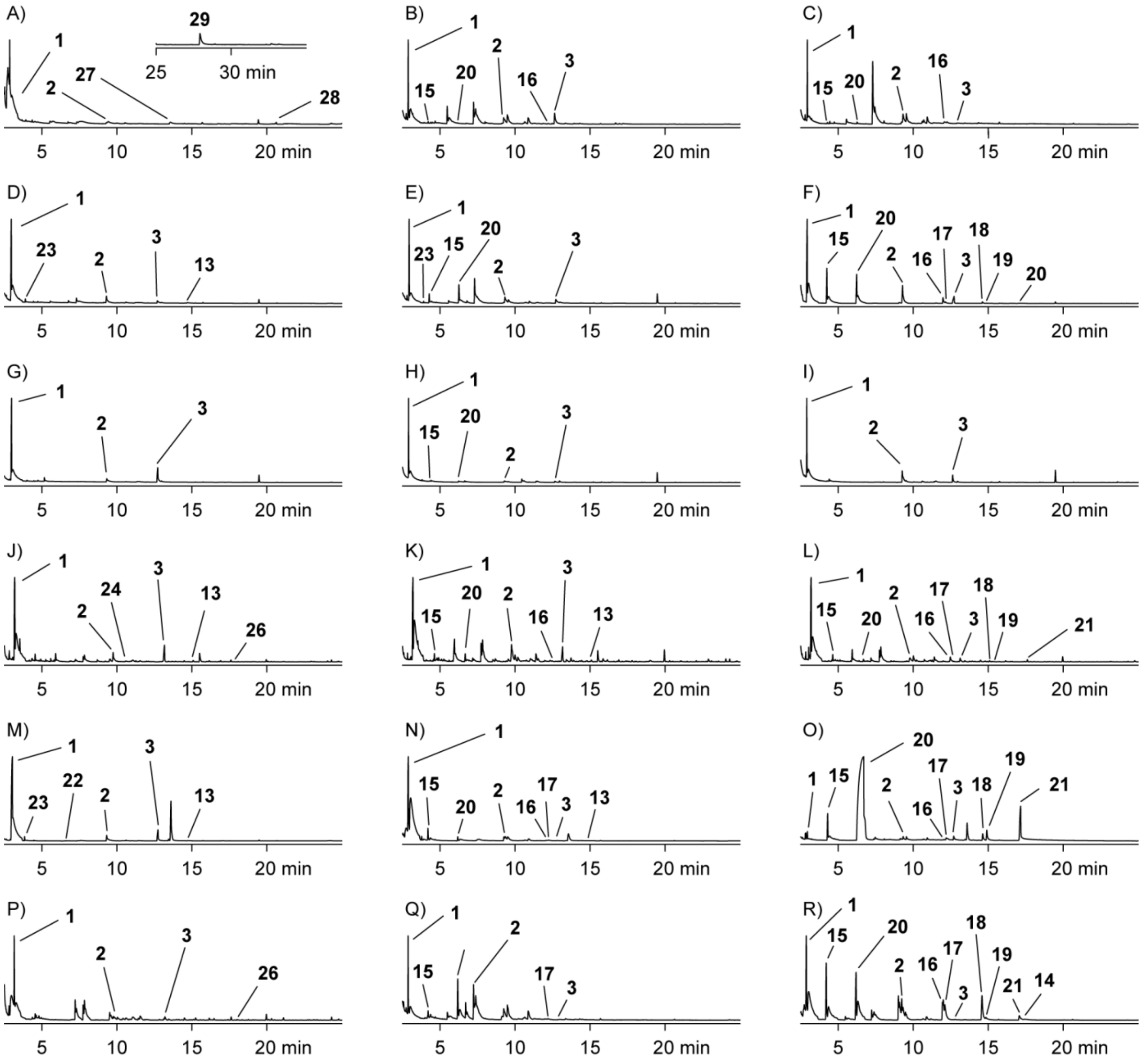

Figure S2: Chromatograms of headspace extracts from *R. denitrificans* DSM 7001 grown on A) 50% MB2216, B) 50% MB2216 + 1 mmol/l Na_2SeO_4, C) 50% MB2216 + 1 mmol/l Na_2SeO_3, from *O. indolifex* DSM 14862 grown on D) 50% MB2216, E) 50% MB2216 + 1 mmol/l Na_2SeO_4, F) 50% MB2216 + 1 mmol/l Na_2SeO_3, from *D. shibae* DSM 16493 grown on G) 50% MB2216, H) 50% MB2216 + 1 mmol/l Na_2SeO_4, I) 50% MB2216 + 1 mmol/l Na_2SeO_3, from *L. alexandrii* DFL-11 grown on J) 50% MB2216, K) 50% MB2216 + 1 mmol/l Na_2SeO_4, L) 50% MB2216 + 1 mmol/l Na_2SeO_3, from *H. phototrophica* DFL-43 grown on M) 50% MB2216, N) 50% MB2216 + 1 mmol/l Na_2SeO_4, O) 50% MB2216 + 1 mmol/l Na_2SeO_3, and from *R. mucosus* DFL-24 grown on P) 50% MB2216, Q) 50% MB2216 + 1 mmol/l Na_2SeO_4, R) 50% MB2216 + 1 mmol/l Na_2SeO_3.

Table S3: Feeding of selenate and selenite to marine bacteria.

Compound[a]	*I*[b]	DSM 17395			DSM 7001			DSM 14862			DSM 16493			DFL-11			DFL-43			DFL-24		
		50% MB2216	+1 mmol/l Na_2SeO_4	+1 mmol/l Na_2SeO_3	50% MB2216	+1 mmol/l Na_2SeO_4	+1 mmol/l Na_2SeO_3	50% MB2216	+1 mmol/l Na_2SeO_4	+1 mmol/l Na_2SeO_3	50% MB2216	+1 mmol/l Na_2SeO_4	+1 mmol/l Na_2SeO_3	50% MB2216	+1 mmol/l Na_2SeO_4	+1 mmol/l Na_2SeO_3	50% MB2216	+1 mmol/l Na_2SeO_4	+1 mmol/l Na_2SeO_3	50% MB2216	+1 mmol/l Na_2SeO_4	+1 mmol/l Na_2SeO_3
Dimethyl disulfide (**1**)	768	xxx	xxx	xxx	xxx	xxx	xxx	xxx	xx	xxx	xxx	xxx	xxx	xxx	xxx	xxx	xxx	xxx	x	xxx	xxx	xxx
S-Methyl propanethioate (**23**)	802							x	x								x					
Dimethyl selenyl sulfide (**15**)	814		xxx	xxx		x	x		x	xxx		x			x	x		xx	xx		xx	xxx
Methylpyrazine	825				x	x	x							x	x	x	x				x	
2-(Methoxymethyl)furan	827					x	x								x	x		x	x		x	x
Furfural	835					x								x	x	x						
2-Furanmethanol	860		xx		xx	xxx	xx							xx	xx	xx					xx	x
Dimethyl diselenide (**20**)	884		xx	xx		x	x		xx	xxx		xx			x	x		xx	xxx		xxx	xxx
Bis(methylthio)methane (**22**)	885																x					
Cyclohexanone	896				x	x								x	x		x	x		xxx	xxx	x
2,5-Dimethylpyrazine	911		xxx		xx	xxx	xxx							x	x	x	x	x	x	xx	xx	xx
2-Acetylfuran	913				xx	xxx	xx							xx	xx	xx	x	xx	x	xxx	xxx	xx
Benzaldehyde	961					x	x			x				x		x			x	xx	x	xx
Dimethyl trisulfide (**2**)	967	x	xx	xxx	xx	xx	xx	xx	x	xxx	xx	xx	xxx	xx	xx	xx	xx	xx	x	xx	xx	xx
4-Methylthio-2-butanone (**24**)	990													x								
2-Acetylthiazole	1016					x	x							x	x	x	x	x		xx	x	x
Benzyl alcohol	1033													x	x	x						
Bis(methylthio)selenide (**16**)	1045		x	x		x	x			xx					x	x		x	x			xx
Methyl methylseleno disulfide (**17**)	1051		x	x						xx						x		x	x		x	xx
S-Methyl methanethiosulfonate (**3**)	1063	xx	xx	x		xx	x	xx	x	xx	xx	x	xx	xx	xx	x	xx	x	x	x	x	x
Acetophenone	1065															x				x		
1,2,4-Trithiolane (**27**)	1088				x																	
1-Undecene	1091																xxx	xx	xx			
Methyl benzoate	1093													x								
Nonanal	1103					x								x		x				x		x
2-Phenylethanol	1111													x	x	x						
Methyl methylthio diselenide (**18**)	1119		x							x						x			x			xx

Methyl (methylthio)methyl disulfide (**13**)	1123							x						x	x		x	x				
Phenylacetone	1127													x		x				x		
Bis(methylseleno)sulfide (**19**)	1127									x						x			x			x
Methyl nicotinate	1137													xx	x	x						
Ethyl benzoate	1170	xx	x					xx	x													
Dimethyl triselenide (**21**)	1203									x						x			xx			x
Decanal	1203				x	x								x		x				x		
Dimethyl tetrasulfide (**14**)	1213		x																			x
Benzothiazol (**26**)	1220													x						x		
Ethyl phenylacetate	1244															x				x		
1,2,4,5-Tetrathiane (**28**)	1350				x																	
Lenthionine (**29**)	1624				xxx																	

[a] Known contaminants such as plasticizers and compounds arising from the agar medium are not mentioned. Relative amounts of compounds were given as follows: x: 0-2%, xx: 2-8%, xxx: >8% of total area in GC. [b] Linear retention index on a HP-5MS fused silicar capillary GC column determined from a homologous series of *n*-alkanes.

References

1. Thole, S.; Kalhoefer, D.; Voget, S.; Berger, M.; Engelhardt, T.; Liesegang, H.; Wollherr, A.; Kjelleberg, S.; Daniel, R.; Simon, M.; Thomas, T.; Brinkhoff, T. *ISME J.* **2012,** *6,* 2229–2244.
2. Petersen, J.; Brinkmann, H.; Berger, M.; Brinkhoff, T.; Päuker, O.; Pradella, S. *Mol Biol Evol.* **2011,** *28,* 1229–1240.
3. González, J. M.; Covert, J. S.; Whitman, W. B.; Henriksen, J. R.; Mayer, F.; Scharf, B.; Schmitt, R.; Buchan, A,; Fuhrman, J. A.; Keine, R. P.; Moran, M. A. *Int. J. Syst. Evol. Microbiol.* **2003,** *53,* 1261–1269.
4. Todd, J. D.; Curson, A. R. J.; Sullivan, M. J.; Kirkwood, M.; Johnston, A. W. B. *PLOS ONE* **2012,** *7,* e35947.
5. Todd, J. D.; Curson, A. R. J.; Kirkwood, M.; Sullivan, M. J.; Green, R. T.; Johnston, A. W. B. *Environ. Microbiol.* **2011,** *13,* 427–438.
6. Shiba, T. *Syst. Appl. Microbiol.* **1991,** *14,* 140–145.
7. Wagner-Döbler, I.; Rheims, H.; Felske, A.; El-Ghezal, A.; Flade-Schröder, D.; Laatsch, H.; Lang, S.; Pukall, R.; Tindall, B. J. *Int. J. Syst. Evol. Microbiol.* **2004,** *54,* 1177–1184.
8. Biebl, H.; Allgaier, M.; Tindall, B. J.; Koblizek, M.; Lünsdorf, H.; Pukall, R.; Wagner-Döbler, I. *Int. J. Syst. Evol. Microbiol.* **2005,** *55,* 1089–1096.
9. Biebl, H.; Pukall, R.; Lünsdorf, H.; Schulz S.; Allgaier, M.; Tindal, B. J.; Wagner-Döbler, I. *Int. J. Syst. Evol. Microbiol.* **2007,** *57,* 1095–1107.
10. Biebl, H.; Tindall, B. J.; Lünsdorf, H.; Allgaier, M.; Wagner-Döbler, I. *Int. J. Syst. Evol. Microbiol.* **2006,** *56,* 821–826.
11. Biebl, H.; Allgaier, M.; Lünsdorf, H.; Pukall, R.; Tindall, B. J.; Wagner-Döbler, I. *Int. J. Syst. Evol. Microbiol.* **2005,** *55,* 2377–2383.
